# Evaluation of the performance of algorithms mapping EORTC QLQ-C30 onto the EQ-5D index in a metastatic colorectal cancer cost-effectiveness model

**DOI:** 10.1186/s12955-020-01481-2

**Published:** 2020-07-20

**Authors:** Mira D. Franken, Anne de Hond, Koen Degeling, Cornelis J. A. Punt, Miriam Koopman, Carin A. Uyl-de Groot, Matthijs M. Versteegh, Martijn G. H. van Oijen

**Affiliations:** 1 University Medical Centre Utrecht, Utrecht University, Cancer Centre, Department of Medical Oncology, P.O. Box 85500, 3508 GA Utrecht, the Netherlands; 2 IT Department, Leiden University Medical Center, Leiden, the Netherlands; 3 Cancer Health Services Research Unit, Faculty of Medicine, Dentistry and Health Sciences, School of Population and Global Health, University of Melbourne, Melbourne, Australia; 4 Department of Medical Oncology, Cancer Center Amsterdam, Amsterdam University Medical Centers, location AMC, University of Amsterdam, Amsterdam, the Netherlands; 5 Institute for Medical Technology Assessment/institute of Health policy and Management, Erasmus University, Rotterdam, the Netherlands

**Keywords:** QLQ-C30, EQ-5D-3L, Quality of life, Utility, Mapping algorithm, Colorectal cancer

## Abstract

**Background:**

Cost-effectiveness models require quality of life utilities calculated from generic preference-based questionnaires, such as EQ-5D. We evaluated the performance of available algorithms for QLQ-C30 conversion into EQ-5D-3L based utilities in a metastatic colorectal cancer (mCRC) patient population and subsequently developed a mCRC specific algorithm. Influence of mapping on cost-effectiveness was evaluated.

**Methods:**

Three available algorithms were compared with observed utilities from the CAIRO3 study. Six models were developed using 5-fold cross-validation: predicting EQ-5D-3L tariffs from QLQ-C30 functional scale scores, continuous QLQ-C30 scores or dummy levels with a random effects model (RE), a most likely probability method on EQ-5D-3L functional scale scores, a beta regression model on QLQ-C30 functional scale scores and a separate equations subgroup approach on QLQ-C30 functional scale scores. Performance was assessed, and algorithms were tested on incomplete QLQ-C30 questionnaires. Influence of utility mapping on incremental cost/QALY gained (ICER) was evaluated in an existing Dutch mCRC cost-effectiveness model.

**Results:**

The available algorithms yielded mean utilities of 1: 0.87 ± sd:0.14,2: 0.81 ± 0.15 (both Dutch tariff) and 3: 0.81 ± sd:0.19. Algorithm 1 and 3 were significantly different from the mean observed utility (0.83 ± 0.17 with Dutch tariff, 0.80 ± 0.20 with U.K. tariff). All new models yielded predicted utilities drawing close to observed utilities; differences were not statistically significant. The existing algorithms resulted in an ICER difference of €10,140 less and €1765 more compared to the observed EQ-5D-3L based ICER (€168,048). The preferred newly developed algorithm was €5094 higher than the observed EQ-5D-3L based ICER. Disparity was explained by minimal diffences in incremental QALYs between models.

**Conclusion:**

Available mapping algorithms sufficiently accurately predict utilities. With the commonly used statistical methods, we did not succeed in developping an improved mapping algorithm. Importantly, cost-effectiveness outcomes in this study were comparable to the original model outcomes between different mapping algorithms. Therefore, mapping can be an adequate solution for cost-effectiveness studies using either a previously designed and validated algorithm or an algorithm developed in this study.

## Background

Measurement of health-related quality of life (HRQoL) with generic questionnaires (e.g. EQ-5D-3L) and disease specific questionnaires (e.g. EORTC QLQ-C30) are of great interest to clinicians and researchers, especially in the context of cost-effectiveness research. In oncology, cost-effectiveness research becomes more important rapidly, as it provides information for decision-makers in establishing the content of the basic benefit package of a health insurance in some countries. Cost-effectiveness outcomes are more often reported in addition to clinical outcome parameters, and the incremental cost per quality adjusted life year (QALY) is generally chosen as primary outcome in cost-effectiveness models [1]. To calculate the total QALYs gained due to treatment, both length and quality of life have to be established. Quality of life can be measured through a generic preference-based quality of life questionnaire such as the commonly used EQ-5D-3L questionnaire, which is requested by some reimbursement authorities [2]. Based on this questionnaire, patient scores are transformed into health-related quality of life utilities, on a scale of 1 - being full health- to 0 - reflecting death (and even negative values reflecting health states worse than death), which can be combined with the duration (survival) of a patient to calculate the QALY [1, 3].

In industry sponsored oncology studies, both the EORTC QLQ-C30 and the EQ-5D questionnaires are often used to capture clinically meaningful changes in quality of life and enable health-economic evaluations [2, 4]. However, the lack of generic preference-based questionnaires in for instance academic clinical studies or clinical registries hamper the calculation of health-related quality of life utilities for cost-effectiveness research. To overcome this issue, researchers often revert to the translation of disease specific quality of life outcomes (such as those captured by QLQ-C30 in oncology) into utilities (such as captured by EQ-5D-3L) using so called ‘mapping algorithms’ for their cost-effectiveness models. Mapping algorithms are regression models developed and tested in specific patient population datasets, which make them ‘sample dependent’. Consequently, Doble et al. [5] demonstrated that in oncology only two out of 10 eligible mapping algorithms, performed sufficiently well in the estimation of utilities (Versteegh et al. using a Dutch tariff for EQ-5D-3L, developed in a multiple myeloma and non-Hodgkin lymphoma dataset, and Longworth et al. for EQ-5D-3L, developed in a multiple myeloma and breast cancer dataset) [5–7]. As shown by Doble et al.*,* QLQ-C30 outcomes between development and validation datasets demonstrated clinically relevant differences on multiple QLQ-C30 dimensions, although congruence of QLQ-C30 outcomes between datasets was not predictive for mapping algorithm performance [5]. Even so, disease related effects could influence the outcomes of mapping algorithms and it has been previously advised to use a mapping algorithm with similar clinical characteristics compared to the sample on which the mapping is to be applied [8]. More recently, Marriott et al. proposed a mapping algorithm developed with a metastatic colorectal cancer (mCRC) patient dataset using an U.K. tariff for EQ-5D-3L [9]. Even so, we question whether the currently available mapping algorithms, which were not all developed with mCRC datasets and an mCRC disease specific algorithm based on a U.K. tariff, are sufficiently suitable to translate QLQ-C30 outcomes to Dutch EQ-5D-3L based utilities for mCRC patients.

Our first objective was to evaluate the accuracy of available mapping algorithms for conversion of QLQ-C30 outcomes to EQ-5D-3L utilities in a population of mCRC patients. Our second objective was to design an mCRC specific mapping algorithm using a Dutch tariff for the conversion of QLQ-C30 outcomes to EQ-5D-3L based utilities. Finally, we evaluated the influence of utility mapping on the incremental cost per QALY gained (ICER) in an existing mCRC cost-effectiveness model [10].

## Methods

### Patient population

The CAIRO3 study is a randomized phase 3 study (NCT00442637) sponsored by the Dutch Colorectal Cancer Group (DCCG), in which mCRC patients with stable disease or better (*n* = 558) following 6 cycles of initial therapy with capecitabine, oxaliplatin and bevacizumab (CAPOX-B). Patients were either randomized to the observation strategy or capecitabine (625 mg/m^2^ orally twice daily continuously) and bevacizumab (7.5 mg/kg intravenously every 3 weeks) (CB) maintenance treatment [11]. Patients completed both the disease specific QLQ-C30 version 3.0 and generic EQ-5D-3L questionnaires every 9 weeks simultaneously [2, 4]. Only patients participating in the completion of QLQ-C30 and EQ-5D questionnaires were selected and all time points were pooled for this study. Descriptive statistics were used for baseline characteristics.

### Questionnaires

The EORTC QLQ-C30 questionnaire version 3.0 comprises 30 questions evaluating quality of life in five functional scales (physical, role, cognitive, emotional and social functioning), three symptom scales (fatigue, pain, nausea and vomiting), global health status and single items for the assessment of symptoms commonly reported by cancer patients (dyspnea, appetite loss, insomnia, constipation, diarrhea and financial difficulties) [4]. QLQ-C30 outcomes were calculated using the EORTC QLQ-C30 scoring manual. After linear transformation and calculation of raw score for the questions ranging not at all (0) to very much (4) for functional and symptom scale scores and very poor (0) to excellent (7) for global health, scale scores range 0 to 100. For functional scales and global health, a high score represents a higher level of functioning, while for the symptoms scales a low outcome represents less symptomatology [12].

The EQ-5D-3L contains 5 questions each addressing a different domain: mobility, self-care, usual activities, pain/discomfort and anxiety/depression. Each of these domains has 3 levels [2]. An EQ-5D-3L based utility is derived from an EQ-5D questionnaire using a country specific value set, i.e. tariff. EQ-5D-3L outcomes in this study were transformed to Dutch and U.K. tariff EQ-5D-3L -based utilities [13, 14].

### Evaluation of existing algorithms

The algorithms by Versteegh et al. and Longworth et al. were initially selected as these performed best in the analysis by Doble and Lorgelly, and is appropriate to the Dutch setting as both can predict Dutch tariff EQ-5D-3L utilities [5, 6]. The mapping algorithm by Marriott et al. was additionally selected as this algorithm was developed in an mCRC patient dataset appropriate to a U.K. setting [8]. All three mapping algorithms were used for prediction of an EQ-5D-3L based utility using concurrently collected EORTC QLQ-C30 outcomes. As the algorithm by Versteegh et al. was based on version 2 of the QLQ-C30 questionnaire, while version 3 was used in the CAIRO3 trial, QLQ-C30 question 1 through 5 were converted into a binary response to fit the mapping algorithm. All algorithms were developed for non-patient level modelling purposes and the performance analysis is therefore focused on their sample means. Some individual level performance characteristics were also used for the mapping algorithms, albeit the well documented suboptimal performance of these algorithms on the individual level in the lower utility ranges. The algorithms were compared to the observed EQ-5D-3L based utilities using the root mean square error (RMSE), mean absolute error (MAE), t-test and Spearman correlation. The data was formatted in STATA. All analyses were performed using R.

### Mapping algorithm design

Methodology according to the MAPS statement was used for developing the mapping algorithm [15]. The mCRC specific mapping algorithms that were developed with commonly used statistical methods and evaluated used 5-fold cross-validation.

Each fold provided a test set in which the trained model, which was developed based on the other 4 folds, could be tested, resulting in 5 estimates for each performance measure.

First, the EQ-5D-3L based utility was regressed on the QLQ-C30 functional and symptom scale scores using a random effects model (RE) with a random intercept: model 1. In a second RE model (model 2), the QLQ-C30 questions were treated as continuous variables and in a third model as dummy variables (model 3). Dummy variables essentially are a redefinition of the four QLQ-C30 answer categories (categories: 1 (no problem at all) to 4 (very much a problem)) and seven categories (categories: 1 (very poor) to 7 (excellent)) for the last two QLQ-C30 questions. For each QLQ-C30 question dummies for outcome categories were regressed on utility prediction. All abovementioned RE models assume a continuous and normal distribution for EQ-5D utilities. Although this assumption is hardly realistic considering the well-studied skewed distribution of utilities, it is by far the most popular form of mapping in the literature and generally performs quite well compared to more complex models [16].

Model 4 is a two-step model, also known as a response mapping model. The advantage of a response mapping model is that it is independent of tariff calculations and it can therefore compute any country utility score for which tariffs are available. First, in model 4, ordered logit regression was used to predict the EQ-5D-3L domain score. An ordered logit model was chosen to preserve the ordering of the categories in the dependent variable.* For this method, input variables were the QLQ-C30 functional scale scores. Secondly, a utility was calculated using the most likely probability method. With the most likely probability method, the probabilities of the EQ-5D-3L response levels (no problem, some problems and severe problems) per EQ-5D domain (mobility, self-care, usual activities, pain/discomfort and anxiety/depression) were predicted based on the QLQ-C30 functional scale scores. The following formulas were used for this:

$$ \boldsymbol{Prob}{\mathbf{1}}_{\boldsymbol{l}\boldsymbol{eve}{\boldsymbol{l}}_{\mathbf{1}}}=\frac{\mathbf{1}}{\mathbf{1}+{\boldsymbol{e}}^{\boldsymbol{EQ}\mathbf{5}\boldsymbol{D}}} $$

Footnote * A multinomial logit model was also developed; however the ordered logit model outperformed the multinomial logit model. Hence, we only report on the ordered logit model in this manuscript.

$$ \boldsymbol{Prob}{\mathbf{2}}_{\boldsymbol{level}\mathbf{2}}=\frac{\mathbf{1}}{\mathbf{1}+{\boldsymbol{e}}^{\boldsymbol{EQ}\mathbf{5}\boldsymbol{D}-\boldsymbol{\kappa}}}-\frac{\mathbf{1}}{\mathbf{1}+{\boldsymbol{e}}^{\boldsymbol{EQ}\mathbf{5}\boldsymbol{D}}} $$

$$ \boldsymbol{Prob}{\mathbf{3}}_{\boldsymbol{l}\boldsymbol{eve}{\boldsymbol{l}}_{\mathbf{3}}}=\mathbf{1}-\boldsymbol{Prob}{\mathbf{1}}_{\boldsymbol{l}\boldsymbol{eve}\boldsymbol{l}\mathbf{1}}-\boldsymbol{Prob}{\mathbf{2}}_{\boldsymbol{l}\boldsymbol{eve}\boldsymbol{l}\mathbf{2}} $$

Where level stands for the EQ-5D-3L response level, EQ. 5D stands for the latent EQ-5D functional or symptom scale score regressed on the QLQ dimensions, ***κ*** stands for the estimated threshold between different response levels. These predicted probabilities were subsequently scored with the EQ-5D scoring system [17].

Model 5 used beta regression to restrict the EQ-5D-3L utilities to the 0,1 interval. The advantage of this method is that it cannot lead to unrealistic utility predictions exceeding 1. However, it will not be able to produce negative utilities. In the current analyses, the number of individuals with negative utilities was so small (0.2%) that this is unlikely to notably affect the results. Moreover, it cannot model values of exactly 1 or 0, so these values were rescaled prior to the mapping. All utilities were first transformed to disutilities. All values ≥1 (which were utilities of 0 or less than 0) were selected to be approximated so that the disutilities would return a value < 1 and thus included in the beta regression. To do so, a standardized value was subtracted from the disutility. All values of exactly 0 (which were utilities of 1) were selected to be adapted so that the disutilities would return values > 0. The standardized transformation applied was: (disutility*(N-1) + 0.5)/N. Nevertheless, the beta distribution is in theory a better approximation of the EQ-5D utility distribution compared to the normal distribution underlying OLS regression, at least in samples with very few health state observations worse than dead. This regression was also conducted on the QLQ-C30 functional scale scores.

The final model (model 6) consisted of a separate equations subgroup approach. In the first step, probabilities are calculated on the basis of a multinomial logistic regression for having a EQ-5D-3L utility score lower than 0.6 (related to scoring ‘extreme problems’ on any EQ-5D-3L dimension [18], higher than 0.6 but lower than 1 and equal to 1. In the next step, RE models are trained on individuals with utility scores lower than 0.6 and higher than 0.6 separately. Finally, the predicted utilities of these two sub-models and of having a 1 are combined with the probabilities from the first step. The advantage of this approach is that it relaxes the assumption of a continuous linear relation between EQ-5D utilities and QLQ-C30 functional and symptom scale scores. Poor health states often adhere to a different (approximate) linear relation with the EQ-5D utilities compared to higher scores, often leading to the overvaluing of low health states in the literature [18].

All models were developed using a backward selection procedure, where non-significant coefficients based on the QLQ-C30 items were removed one-by-one (cut-off value *p* = 0.05) until all coefficients were at or below the cut-off value. Except for model 4 and 6 (in part), backward selection was performed to minimize the mapping algorithm length without compromising the model performance, which has previously been done by others [6, 7]. In a second step, non-logical coefficients were removed. Non-logical coefficients were defined as coefficients that carried an incongruous sign, for example a coefficient for nausea leading to a better utility when one would expect a reduction in the assigned utility. Random effects with cluster robust standard errors were introduced to correct for multiple responses from one patient for all OLS models (models 1, 2, 3, and 6 in part). The beta, ordered logit and multinomial logit regressions (models 4, 5 and 6 in part) used normal standard errors as there were no cluster robust standard errors available for these methods.

### Validation of the developed mapping algorithms

After development of the six mapping algorithms using each of the five training data sets consecutively, the algorithms were tested in the corresponding folds. Performance of the algorithms was reported as mean predicted utility, the root mean squared error (RMSE) and mean absolute error (MAE). The RMSE will give a better insight into the performance of the mapping algorithm alongside MAE, as it is more sensitive to outliers and hence helps identify the mapping algorithm with the least extreme deviations between predicted and observed values. The resulting algorithms were analyzed for logical consistency using scatter plots comparing observed and predicted utilities, i.e. worse outcomes of the observed EQ-5D-3L based utility also lead to worse outcomes in the predicted utilities with the six methods described above. Lastly, Spearman correlation coefficients and t-tests were used to illustrate the performance of the various algorithms. The model of preference was selected based on best fit: smallest value for RMSE, MAE and highest value for the Spearman correlation.

Performance of the mapping algorithms based on QLQ-C30 functional scale scores, developed with OLS, response mapping, beta regression and the separate equations model, were tested on incomplete QLQ-C30 questionnaires. Quality of life functional scale scores (e.g. physical functioning) can be calculated with a minimal completion of half of the questions included in the QLQ-C30 questionnaires [12]. Incomplete questionnaires, for which functional scale scores calculations remained possible and with a concurrently collected EQ-5D-3L, were selected to test mapping algorithm performance with those algorithms based on functional scale scores. No imputations were performed on QLQ-C30 questionnaires. Results were compared with concurrently collected EQ-5D-3L questionnaires. Outcomes were compared with observed utilities as previously described.

### Algorithm influence on cost-effectiveness model outcomes

The influence of the mapping algorithms on the primary outcome, the incremental cost per QALY gained (ICER), was evaluated using a Dutch cost-effectiveness model comparing CB maintenance and observation following 6 cycles of first line CAPOX-B for patients with mCRC. For this purpose, a discrete event simulation model, developed in AnyLogic (multi-method simulation software, v.8.2.3, The AnyLogic Company (Chicago, IL, USA) was used for the current analysis [19]. ICERs comparing CB maintenance and observation were calculated for 1) observed EQ-5D-3L based utilities as was done in the original study, 2) utilities obtained with the mapping algorithm developed by Versteegh et al. [6] (mapping algorithm for a Dutch tariff conversion), 3) utilities obtained with the mapping algorithm developed by Longworth et al. using a Dutch tariff and 4) utilities obtained with the preferred mapping algorithm developed in this study (model 1). The mapping algorithm developed by Marriott et al. [9] uses a U.K. tariff conversion and was therefore not included. Only concurrently collected EQ-5D and QLQ-C30 observations during either maintenance treatment and observation, defined as the first health-state, were used in this analysis. Utilities in subsequent health-states (re-introduction of therapy, salvage therapy, death) were derived from literature as these could not be derived from the CAIRO3 study [10].

A total of 10,000 hypothetical patients per treatment strategy were simulated for a patient-level outcome calculation. Subsequently, a probabilistic analysis was performed to calculate the ICERs with a 95% confidence interval based on 10,000 samples. To reflect parameter uncertainty in the probabilistic analysis, distributions for the utilities were defined according to the method of moments using the mean and a standard error for each of the utilities derived from the selected mapping algorithms in line with the original cost-effectiveness evaluation of the CAIRO3 study. With the exception of the uncertainty around utilities only, distributions for the other parameters, such as costs, health-state transitions, were defined as in the original cost-effectiveness evaluation of the CAIRO3 study [10].

## Results

From a total of 2440 observations, 1905 concurrently collected, complete QLQ-C30 and EQ-5D-3L questionnaires were included in this analysis. The concurrent observations were obtained from 473 patients enrolled in the CAIRO3 study (238 patients in the observation arm and 235 patients in the maintenance treatment arm). In Table 1, characteristics of the QLQ-C30 and EQ-5D data set are presented. The distribution of EQ-5D based utilities can be viewed in Additional File 1. Incomplete QLQ-C30 or EQ-5D-3L questionnaires were excluded for mapping algorithm development. For the purpose of the mCRC specific mapping algorithm design, we randomly divided the data in 5 folds (*n* = 381 each).

**Table 1:** Patient characteristics for concurently collected EQ-5D and QLQ-C30 questionnaires

		Complete *N* = 1905
Age (years)		64 (8.4)
Male gender (%)		69
EQ-5D-3L^a^	N	1905
	Mobility 1/2/3 (%)	57.9/41.8/0.3
	Self-cae 1/2/3 (%)	93.4/6.1/0.4
	Usual activities 1/2/3 (%)	57.5/38.5/3.9
	Pain/discomfort 1/2/3 (%)	60.2/38.4/1.4
	Depression/anxiety 1/2/3 (%)	77.2/21.8/1
	EQ-5D utility, mean (SD)	0.834 (0.171)
	EQ-5D range	− 0.134 to 1
QLQ-C30 v.3.0	Questionnaires, N	1905
	Physical functioning, mean (SD)	82.681 (17.195)
	Role functioning, mean (SD)	76.947 (24.218)
	Emotional functioning, mean (SD)	85.744 (15.829)
	Cognitive functioning, mean (SD)	89.221 (15.294)
	Social functioning, mean (SD)	86.177 (18.718)
	Global health, mean (SD)	74.711 (17.464)
	Fatigue, mean (SD)	24.205 (20.059)
	Nausea/vomiting, mean (SD)	4.234 (11.286)
	Pain, mean (SD)	13.508 (20.705)
	Dyspnea, mean (SD)	10.866 (19.061)
	Insomnia, mean (SD)	15.083 (22.297)
	Appetite, mean (SD)	9.729 (19.651)
	Constipation, mean (SD)	6.824 (15.917)
	Diarrhea, mean (SD)	10.569 (19.363)
	Financial difficulties, mean (SD)	6.229 (15.978)
Concurent EQ-5D and incomplete QLQ-C30 with retainment of functional scale scores, N^b^		120

^a^Percentages at level 1, 2 and 3 represent no problems at all, some problems and extreme problems, respectively

^b^Patient characteristics for concurently collected incomplete QLQ-C30 questinnaires available in Additional file 3

### Performance of existing mapping algorithms on an mCRC dataset

The mean observed utility based on completed EQ-5D-3L questionnaires of the mCRC dataset included in this analysis was 0.834 ± sd: 0.171 (Dutch tariff) and 0.803 ± sd: 0.197 (U.K. tariff). The algorithm by Versteegh et al. resulted in a mean utility of 0.866 ± 0.135 with a Spearman correlation of 0.76 (*p* < 0.01) (Table 2). The algorithm by Longworth et al. resulted in a mean utility of 0.835 ± 0.127 and 0.810 ± 0.152, with a Spearman correlation of 0.77 and 0.79, for the Dutch tariff and the U.K. tariff respectively. The algorithm by Longworth for Dutch tariff performed very well and was not significantly different compared to observed utilities (*p* = 0.687). The algorithm by Marriott et al. (U.K. tariff) resulted in a mean utility of 0.813 ± sd:0.185 with a Spearman correlation of 0.75 (*p* < 0.01) (Table 2).

**Table 2:** Utility, observed and predicted, for all patients with complete questionnaires (*n* = 1905)

	Mean utility	SD	Min.	Max.	RMSE	MAE	Spearman correlation	*p*-value
Observed utility (Dutch tariff)	0.834	0.171	−0.134	1	-	-	-	-
Observed utility (U.K. tariff)	0.803	0.197	− 0.239	1	-	-	-	-
Predicted utility (Versteegh (6))	0.866	0.135	−0.298	0.978	0.113	0.080	0.76	< 0.001*
Predicted utility (Longworth (7) (Dutch tariff)	0.835	0.127	−0.088	0.959	0.106	0.078	0.77	0.687*
Predicted utility (Longworth (7) (U.K. tariff)	0.810	0.152	−0.307	0.955	0.114	0.085	0.79	0.026**
Predicted utility (Marriott (9))	0.813	0.185	−0.159	1.061	0.122	0.089	0.75	0.001**

*p-value tested against Dutch tariff ** *p*-value tested against U.K. tariff; *p*-values result from a t-test

### Design and validation of a new mapping algorithm on a mCRC dataset

Algorithm coefficients for the RE based algorithms are presented in Tables 3 (model 1), 4 (model 2) and 5 (model 3). These algorithms concern the RE model with QLQ-C30 functional scale scores (model 1), RE model with QLQ-C30 question outcomes as continuous variable (model 2) and RE model with the QLQ-C30 questions as dummy variables (model 3). The ordered logit regressions for prediction of the EQ-5D-3L based utility (model 4) can be viewed in the Additional file 2: Tables 1-3. The beta regression (model 5) output can be found in Table 6 and the separate equations subgroup approach model (model 6) in Additional file 2 Tables 4-6.

**Table 3:** Regression results for model 1: EQ-5D-3L based utility values on QLQ-C30 domain scores

Variable	Coefficient (SD)	t-value	*p*-value	95% CI
Constant	0.2993 (0.027)	10.940	< 0.001	[0.246, 0.353]
Physical functioning	0.0021 (0.000)	7.949	< 0.001	[0.002, 0.003]
Role functioning	0.0011 (0.000)	5.738	< 0.001	[0.001, 0.001]
Emotional functioning	0.0025 (0.000)	10.901	< 0.001	[0.002, 0.003]
Cognitive functioning	0.0005 (0.000)	2.279	0.023	[0.000, 0.001]
Social functioning	0.0006 (0.000)	2.814	0.005	[0.000, 0.001]
Symptom scale: Pain	−0.0023 (0.000)	−13.519	< 0.001	[− 0.003, − 0.002]
Symptom scale: Insomnia	− 0.0005 (0.000)	−3.166	0.002	[− 0.001, 0.000]

Symptom scale nausea and vomiting was removed as non-logical coefficient

*p*-values result from a t-test

**Table 4:** Regression results for model 2: EQ-5D-3L based utility values QLQ-C30 questions as continuous variables

Variable	Coefficient (SD)	t-value	*p*-value	95% CI
Constant	1.340 (0.015)	86.755	< 0.001	[1.310, 1.370]
QLQ3	− 0.031 (0.006)	−5.553	< 0.001	[− 0.042, − 0.020]
QLQ5	− 0.077 (0.011)	−7.056	< 0.001	[− 0.098, − 0.055]
QLQ6	− 0.048 (0.005)	−9.660	< 0.001	[− 0.057, − 0.038]
QLQ9	− 0.053 (0.006)	−9.305	< 0.001	[− 0.064, − 0.042]
QLQ11	− 0.018 (0.005)	−3.686	< 0.001	[− 0.027, − 0.008]
QLQ19	− 0.021 (0.007)	− 3.150	0.002	[− 0.033, − 0.008]
QLQ22	− 0.021 (0.005)	−4.126	< 0.001	[− 0.031, − 0.011]
QLQ23	− 0.025 (0.006)	− 4.010	< 0.001	[− 0.038, − 0.013]
QLQ24	− 0.040 (0.007)	−6.113	< 0.001	[− 0.053, − 0.027]
QLQ26	− 0.026 (0.006)	−4.546	< 0.001	[− 0.037, − 0.015]
QLQ28	− 0.012 (0.006)	− 1.913	0.056	[− 0.025, 0.000]

QLQ15 (vomiting) was removed as non-logical coefficient

*p*-values result from a t-test

**Table 5:** Regression results for model 3: EQ-5D-3L based utilities on QLQ-C30 questions as dummy variables

Variable	Coefficient (SD)	t-value	*p*-value	95% CI
Constant	0.966 (0.006)	158.046	< 0.001	[0.954, 0.978]
QLQ1_quite a bit	− 0.020 (0.009)	−2.142	0.032	[− 0.038, − 0.002]
QLQ2_a little	− 0.014 (0.006)	− 2.324	0.020	[− 0.026, − 0.002]
QLQ3_a little	− 0.028 (0.008)	− 3.670	< 0.001	[− 0.043, − 0.013]
QLQ3_quite a bit	− 0.046 (0.014)	−3.231	0.001	[− 0.074, − 0.018]
QLQ5_ a little	−0.065 (0.013)	−4.903	< 0.001	[− 0.091, − 0.039]
QLQ5_ quite a bit	− 0.225 (0.037)	−6.097	< 0.001	[− 0.297, − 0.153]
QLQ6_ a little	− 0.047 (0.007)	− 6.978	< 0.001	[− 0.06, − 0.034]
QLQ6_ quite a bit	−0.076 (0.011)	−6.780	< 0.001	[− 0.098, − 0.054]
QLQ6_ very much	− 0.259 (0.020)	− 13.215	< 0.001	[− 0.297, − 0.22]
QLQ9_ a little	−0.068 (0.007)	−10.468	< 0.001	[− 0.081, − 0.055]
QLQ9_quite a bit	− 0.099 (0.012)	− 8.327	< 0.001	[− 0.123, − 0.076]
QLQ9_very much	−0.168 (0.023)	−7.383	< 0.001	[− 0.213, − 0.123]
QLQ18_very much	− 0.058 (0.020)	− 2.933	0.003	[− 0.097, − 0.019]
QLQ19_quite a bit	−0.062 (0.014)	− 4.475	< 0.001	[− 0.089, − 0.035]
QLQ22_a little	− 0.027 (0.006)	− 4.251	< 0.001	[− 0.039, − 0.014]
QLQ22_quite a bit	− 0.046 (0.012)	− 3.733	< 0.001	[− 0.07, − 0.022]
QLQ23_quite a bit	− 0.060 (0.018)	− 3.294	0.001	[− 0.096, − 0.024]
QLQ23_ very much	−0.211 (0.049)	−4.338	< 0.001	[− 0.306, − 0.115]
QLQ24_a little	− 0.045 (0.007)	− 6.117	< 0.001	[− 0.059, − 0.03]
QLQ24_quite a bit	−0.108 (0.020)	−5.264	< 0.001	[− 0.148, − 0.068]
QLQ24_very much	− 0.213 (0.035)	− 6.039	< 0.001	[− 0.283, − 0.144]
QLQ26_a little	−0.033 (0.007)	−4.874	< 0.001	[− 0.047, − 0.02]
QLQ26_quite a bit	− 0.068 (0.016)	− 4.385	< 0.001	[− 0.099, − 0.038]
QLQ28_ very much	−0.056 (0.022)	−2.603	0.009	[− 0.098, − 0.014]

*p*-values result from a t-test

**Table 6:** Beta regression results for model 5: EQ-5D-3L based disutility values on QLQ-C30 domain scores

Variable	Coefficient (SD)	t-value	*p*-value	95% CI
Constant	2.081 (0.210)	9.898	< 0.001	[1.669,2.493]
Global health	−0.004 (0.002)	−1.892	0.058	[−0.007,0]
Physical functioning	−0.018 (0.002)	−8.269	< 0.001	[−0.022,-0.014]
Role functioning	−0.010 (0.002)	−6.125	< 0.001	[−0.013,-0.007]
Emotional functioning	−0.015 (0.002)	−8.479	< 0.001	[−0.019,-0.012]
Cognitive functioning	−0.005 (0.002)	−3.063	0.002	[−0.009,-0.002]
Symptom scale: pain	0.014 (0.001)	9.939	< 0.001	[0.011,0.017]
Symptom scale: insomnia	0.005 (0.001)	4.119	< 0.001	[0.002,0.007]
Symptom scale: financial	0.007 (0.001)	4.448	< 0.001	[0.004,0.009]

Symptom scale nausea and vomiting was removed as non-logical coefficient

*p*-values result from a t-test

**Fig. 1:**
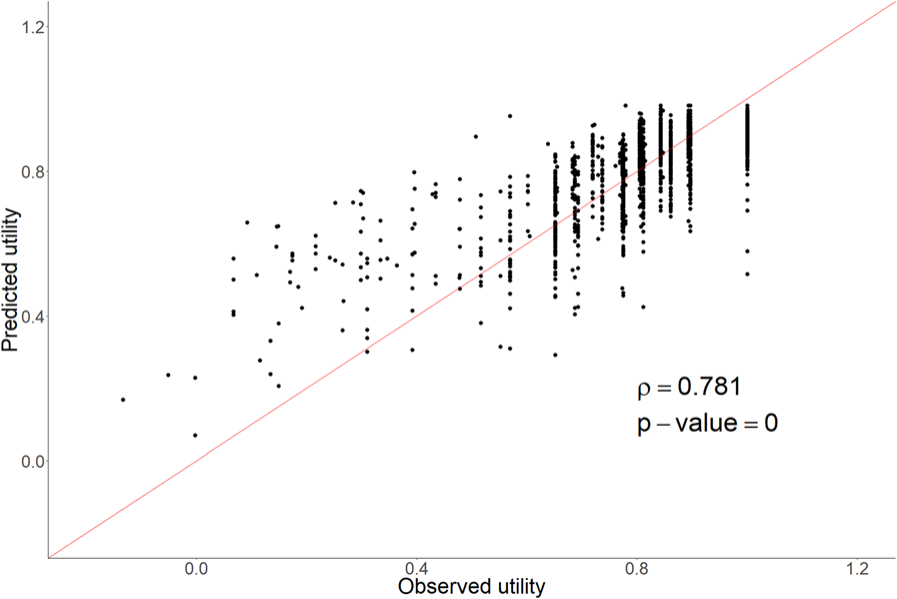
Correlation of observed versus predicted utility for model 1. Observed utility values were based on the EQ-5D-3L questionnaire and regressed on the QLQ-C30 functional and symptom scale scores

Observed and mean predicted utility resulting from the six developed mapping algorithms are presented in Table 7. The mean observed utility was 0.834 ± 0.171, while the mean predicted utilities for model 1 to 6 were nearly identical, 0.832 ± 0.134, 0.832 ± 0.134, 0.833 ± 0.133, 0.830 ± 0.145, 0.838 ± 0.156 and 0.834 ± 0.138, respectively. A utility prediction drawing close to the observed utility was achieved in all models. Differences between observed and predicted utilities were non-significant. The lowest RMSE and MAE was achieved by model 1 (RMSE 0.098, MAE 0.072) and model 4 (RMSE 0.098, MAE 0.072). Note that comparable to the Longworth algorithm, model 4 is an algorithm for EQ-5D response prediction and is thus independent of country tariff. For the purpose of comparison between model performance, a Dutch tariff was applied to the Longworth algorithm and model 4. Mapping algorithms based on functional scale scores are more forgiving towards incomplete questionnaires, as quality of life functional scale scores (e.g. physical functioning) can be calculated with a minimal completion of half of the questions included in the QLQ-C30 questionnaires. Performance of all newly developed mapping algorithms using QLQ-C30 functional scale scores (model 1, 4, 5 and 6), were additionally tested in incomplete QLQ-C30 questionnaires for which functional scale scores could still be calculated for which EQ-5D outcomes were concurrently available (*n* = 120). Patient characteristics of incomplete questionnaires are presented in Additional file 3. The mean observed utility in 120 incomplete QLQ-C30 questionnaires was 0.760 ± 0232. The best predicted mean utilities were 0.767 ± 0.177, 0.756 ± 0.222, 0.764 ± 0.222, for model 1, model 4 and model 5 respectively (Table 8). The lowest RMSE an MAE were achieved for model 1, which was chosen as preferred model. The algorithm based on the QLQ-C30 functional scale scores (preferred model) was regarded effective based on correlation between observed and mapped utilities (Fig. 1).

**Table 7:** Mean, standard deviation, minimum and maximum of utility values, RMSE and MAE for the predicted utilities (*p*-values result from a t-test)

	Observed utility NL	Model 1 RE functional scale scores^a^	Model 2 RE: continuous	Model 3 RE: dummy variables	Model 4 Ordered logit	Model 5 Beta regression	Model 6 Separate equations
Mean	0.834	0.832	0.832	0.833	0.830	0.838	0.834
St.Dev	0.171	0.134	0.134	0.133	0.145	0.156	0.138
Min	−0.134	0.069	−0.055	0.057	−0.206	0.034	0.178
Max	1	0.982	0.969	0.966	0.975	0.959	0.990
RMSE	–	0.098	0.098	0.103	0.098	0.106	0.100
MAE	–	0.072	0.075	0.077	0.072	0.081	0.070
Spearman correlation	–	0.781	0.780	0.779	0.786	0.774	0.787
*p*-value	–	0.564	0.501	0.615	0.125	0.108	0.943

^a^Preferred model

*p*-values result from a t-test

**Table 8:** Mean, standard deviation, minimum and maximum of utility values, RMSE and MAE for the predicted utilities for incomplete questionnaires (n = 120) with algorithms using domain scores for utility prediction (model 1, 4, 5 and 6)

	Observed utility NL	Model 1 RE functional scale scores*	Model 4 Ordered logit	Model 5 Beta regression	Model 6 Separate equations subgroup approach
Mean	0.760	0.764	0.756	0.764	0.767
St.Dev	0.232	0.177	0.222	0.222	0.178
Min	−0.086	0.108	−0.178	0.032	0.232
Max	1	0.982	0.971	0.959	0.985
RMSE	–	0.128	0.139	0.131	0.128
MAE	–	0.085	0.086	0.088	0.085
Spearman correlation	–	0.843	0.827	0.828	0.827
p-value	–	0.734	0.945	0.751	0.723

*p*-values result from a t-test

Figures depicting the error of predicted utilities compared to the observed utilities for each algorithm are available in the Additional file 4: Figs. 2 and 3. As is well documented in the literature [18], all mapping algorithms show overestimation of lower utilities and underestimation of high utilities.

### Algorithm influence on ICERs in a mCRC cost-effectiveness model

**Fig. 2:**
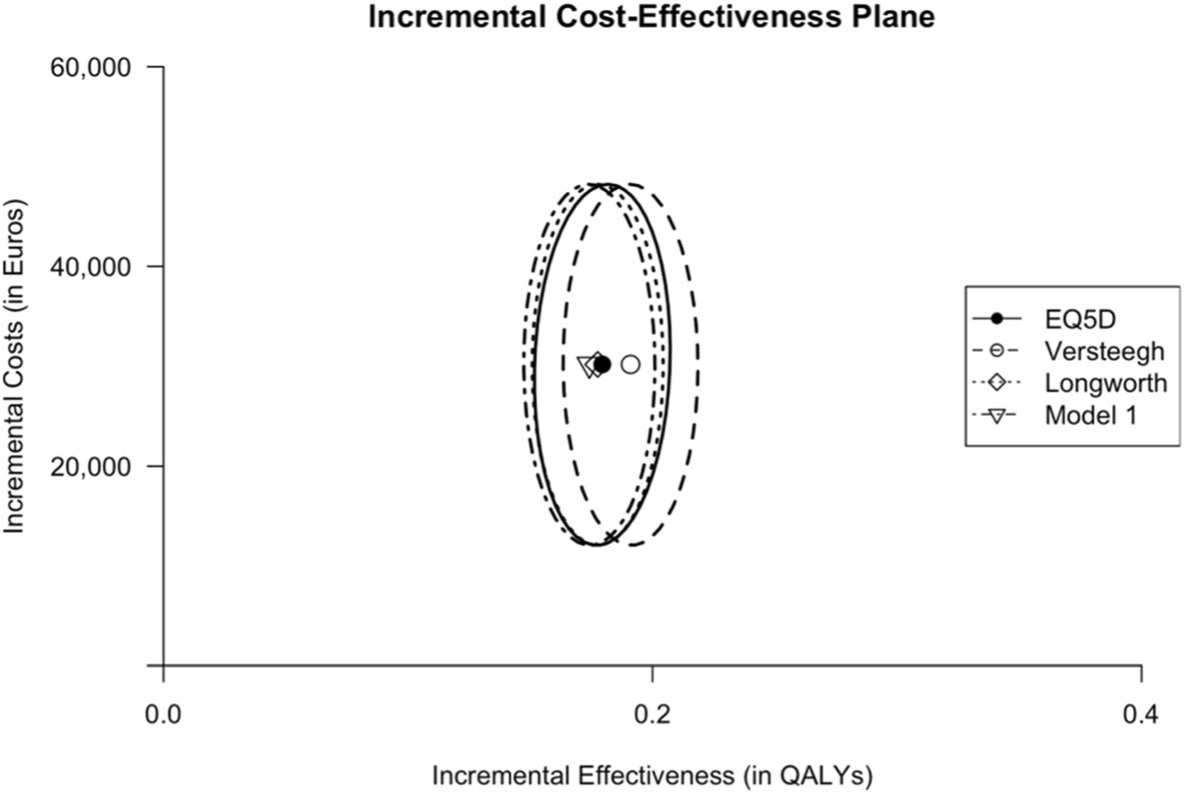
Incremental cost-effectiveness plans for observed and predicted utilities. Incremental cost-effectiveness planes comparing the effect of using observed EQ-5D-3L utility, the mapping algorithm by Versteegh et al.*,* the mapping algorithm by Longworth et al (based on Dutch tariff)*.* and predicted utility based on the preferred model (model 1 on OLS algorithm on QLQ-C30 functional scale scores). Ellipses represent the 95% confidence interval

The influence of the mapping algorithms on the ICER, was tested in an existing Dutch cost-effectiveness model comparing two different treatment strategies (CB maintenance versus observation following 6 cycles of first line CAPOX-B) in an mCRC patient population. For the first health state in this cost-effectiveness model, utilities were estimated using a total of 1654 observations (709 observations for 223 patients in the observation arm and 945 observations for 225 patients in the maintenance arm), utilities of subsequent health states (first progression and theirafter) were derived from literature as was done in the original cost-effectiveness study. The ICERs presented in Table 9 were obtained with 1) observed EQ-5D-3L based utilities, 2) utilities obtained with the mapping algorithm developed by Versteegh et al., 3) utilities obtained with the mapping algorithm developed by Longworth et al using a Dutch tariff and 4) utilities obtained with the preferred model 1. The calculated ICER based on observed utilities in this analysis was €168,048/QALY. Previously developped mapping algorithm by Versteegh et al. compared to the observed EQ-5D-3L based utility lead to a negative ICER difference in the point estimate of €10,140 per QALY gained, while a positive difference of €5094 and €1765 was shown for the preferred algorithm (model 1) and the Longworth algorithm, respectively (Fig. 2).

**Table 9:** Effect of utility mapping on the incremental cost/QALY in a discrete event simulation model

	Utility Observation	Utility CB Maintenance	Incremental costs (€)	Incremental QALYs	ICER (€/QALY)	ICER difference
EQ-5D-3L based utility	0.829 (SE 0.0080)	0.839 (SE 0.0055)	30,163	0.179	168,048	
Versteegh utility (6)	0.876 (SE 0.0052)	0.875 (SE 0.0040)	30,163	0.191	157,908	- €10,140
Longworth (Dutch) utility(7)	0.840 (SE 0.0052)	0.843 (SE 0.0038)	30,163	0.178	169,812	€1765
Model 1^a^	0.836 (SE 0.0053)	0.837 (SE 0.0041)	30,163	0.174	173,141	€5094

*CB* Capecitabine and bevacizumab

^a^based on the OLS domain scores

## Discussion

We have shown that the previously developed algorithm by Versteegh et al. and Marriott et al. for conversion of the disease-specific questionnaire EORTC QLQ-C30 into EQ-5D-3L based utilities resulted in a statistically significant difference between predicted and observed utilities. Still, the existing algorithms performed well as the mean predicted utilities drew close to the mean observed utilities (mean differences between the observed and respectively the mapped utilities by Versteegh et al., Longworth et al. and Marriott et al. were 0.03, 0.001 and 0.01 for the Dutch tariff EQ-5D utilities). No significant difference between, observed and predicted utilities were seen with the algorithm developed by Longworth et al. Even though the predicted utilities calculated with the algorithms by Versteegh et al. and Marriott et al. were significantly different, the outcome differences were not considered clinically meaningful. Previously, the minimal clinically relevant difference in utility for cancer patients was found to range 0.08–0.16, although this difference might vary per patient population [20, 21]. Moreover, patients with different cancers types and stages of disease experience different symptoms and may thus respond differently on the QLQ-C30 functional scale scores [8]. In contrast, as was previously shown by Doble et al. disease severity is more likely to drive EQ-5D estimation based on QLQ-C30, and less by the cancer type [5]. Moreover, several studies developed condition-specific instruments, such as the EORTC QLU-C10D to derive health-related quality of life utilities, which might be more sensitive to disease-specific effects and in theory be preferred over EQ-5D. However, one can question whether these condition-specific instruments outperform EQ-5D [22–24]. Finally, with the emergence of novel treatment strategies in cancer treatment, such as immunotherapy, one could hypothesize a different value of QLQ-C30 functional scale or symptom scores, which could affect mapping outcomes.

Nevertheless, we pursued a better fitting algorithm for the mCRC patient population. All developed models demonstrated improved utility prediction ability with non-significant differences between observed and predicted utilities, although we acknowledge that the performance of the models developed in this study are not tested in a truly external dataset (as the models taken from the literature). Importantly, with the commonly used statistical methods to develop mapping algorithm, we did not succeed in the development of a better performing mapping algorithm. In case a mapping algorithm would be selected from our study, we would suggest the use of the RE model based on QLQ-C30 functional scale scores (model 1). This model provided the benefit of utility prediction for incomplete QLQ-C30 questionnaires (for which functional scale scores could be calculated), while retaining a good performance if tested on incomplete QLQ-C30 questionnaires. QLQ-C30 outcome conversion into EQ-5D-3L based utilities (Dutch tariff) could therefore be performed with the following algorithm, developed on functional scale scores *(model 1)*:

$$ {\displaystyle \begin{array}{c} EQ\hbox{-} 5{D}_{utility}= 0.2993+ 0.0021\ast physical\ functioning\ score+ 0.0011\ast role\ functioning\ score+ 0.0025\ast \\ {} emotional\ functioning\ score+ 0.0005\ast cognitive\ functioning\ score+ 0.0006\ast social\ functioning\\ {} score+ pain\ score\ast \hbox{-} 0.0023+ insomnia\ score\ast \hbox{-} 0.0005.\end{array}} $$

The main purpose of mapping algorithms is to convert disease specific quality of life data into utilities for the purpose of cost-effectiveness research, if utilities cannot directly be derived from the dataset. We investigated the influence of a mapping algorithm on a cost-effectiveness model evaluating CB maintenance treatment compared to observation in mCRC patients. We demonstrated that the use of mappings results in comparable outcomes when used in a cost-effectiveness model. The newly developed algorithm slightly underperformed compared to the previously developed algorithm by Longworth et al. (ICER differences between in CEA using observed utilities and mapping: €1765/QALY gained for the Longworth et al. mapping and €5094 /QALY gained for the preferred model 1 in this study)*.* An ICER difference of -€10,140/QALY gained was seen if compared to the Versteegh et al. mapping. Disparities were explained by small differences in incremental QALY estimation between treatment arms. The algorithm by Versteegh et al. and Longworth et al. slightly overestimated the utilities in both study arms; while the preferred model algorithm (model 1) overestimated the utilities in the observation arm and underestimated the utilities in the CB maintenance arm. Nevertheless, the Longworth algorithm outperformed our preferred model algorithm in this cost-effectiveness model. In a model with more pronounced utility differences, the impact of the chosen mapping algorithm might be different due to case mix effects. The good performance of the Longworth algorithm in this study is remarkable, as this algorithm had not been developed on colon cancer patients, and was estimated on an entirely different sample. Hence, its good performance, especially relative to the within-sample validation of the algorithm we developed, shows the usefulness of this flexible algorithm. Its performance raises the question if similarity of symptoms and severity of symptoms between the development sample and the application sample might not be of greater importance than type of cancer or tumor. While this study seems to suggest that indeed tumor type is less relevant, such a statement must be made with caution: many mapping algorithms, including the one by Versteegh et al., use only a selection of items of the QLQ-C30. As a consequence, out of sample prediction in patients with other cancer types with specific symptoms not captured by the included items might be complicated.

A strength of this study was the use of multiple statistical methods which enabled us to evaluate and select the best-performing algorithm, while also considering convenience in use. Furthermore, the analyses were conducted on a large population of patients, with a total of 1905 completed questionnaires. As previously mentioned, the algorithm by Versteegh et al. and the algorithm by Longworth et al. were not developed or validated in mCRC patient populations [6, 7]. Only, the algorithm by Marriott et al. was developed and tested in an mCRC patient population using a U.K. tariff for EQ-5D-3L [9]. Patients with different cancers types and stages of disease experience different symptoms and might thus respond differently on the QLQ-C30 domains functional scale scores. Thus, the most applicable algorithm in terms of cancer type and disease stage, should be applied for utility prediction, although it has previously been shown to be more dependent of disease severity than cancer type [5]. Of note, another colorectal cancer specific mapping algorithm estimating EQ-5D-5L values using a U.K. tariff was previously developed [25, 26]. However, this mapping algorithm could not be tested and validated with the EQ-5D-3L values in our dataset, as this would require an additional mapping of EQ-5D-3L to EQ-5D-5L and we consequently would not been able to separate performance of the mapping algorithm due to differences in utilities. Currently, the EQ-5D-5L questionnaire is increasingly being adopted in clinical trials as it is regarded more sensitive to health effects and reduce ceiling effects [27]. Further research on mapping of QLQ-C30 outcomes towards EQ-5D-5L is therefore necessary.

The mapping algorithm was developed using a single sample, in which completed questionnaires were assigned to one of five folds that functioned as hold-out sample, which may be regarded as limitation of this study. Inevitably, the training and test datasets therefore contain comparable patients, who completed the quality of life questionnaires under similar circumstance. Preferably, validation of the developed algorithms should have occurred in another sample containing mCRC patient data on both the QLQ-C30 and the EQ-5D-3L questionnaires. Another limitation to this study, is the use of different time-points. The regression algorithms accounted for the panel data structure where possible through the use of random effects models. However, it has previously been shown that colorectal cancer patients continue to report high quality of life during the course of their disease [28–31]. Nonetheless, significant and clinically relevant changes in quality of life occur in the palliative stage of the disease, especially in the last few months of life a decline in quality of life has been demonstrated [32]. Therefore, it may be hypothesized that this could also apply for different time-points within a trial during which different dimensions of health are affected. The models developed in this study, are especially sensitive to this issue.

## Conclusion

We have developed a QLQ-C30 to EQ-5D-3L mapping algorithm on a mCRC patient population with predicted utilities drawing close to the observed utilities. However, the mapping algorithm did not outperform existing mapping algorithms, especially compared with the response mapping algorithm by Longworth et al. Moreover, external validation of our preferred mapping algorithm remains desirable. The choice of mapping algorithm might only have a small impact on the predicted utility and cost-effectiveness, as was illustrated in the case study. Nonetheless, for studies only including disease-specific quality of life questionnaires, our results show that mapping is an adequate solution to obtain utility estimates for use in cost-effectiveness analysis for mCRC patients, using either our newly developed mapping algorithm or one of the existing algorithms used in this study.

## Supplementary information

**Additional file 1.** Histogram of EQ-5D-3L based utilities of 1905 observations.

**Additional file 2: Table 1.** Ordered logit regression (model 4) results for QLQ-C30 domain scores on EQ-5D-3L domain. **Table 2.** Ordered logit regression (model 4) results for QLQ-C30 domain scores on EQ-5D-3L domain. **Table 3.** Ordered logit regression (model 4) results for QLQ-C30 domain scores on EQ-5D-3L domain. **Table 4.** Separate equations subgroup approach (model 6) results for QLQ-C30 domain scores on EQ-5D-3L utility of i) < 0.6, ii) ≥ 0.6 and < 1 and iii) 1. **Table 5.** Regression results (model 6) for EQ-5D-3L based utility values < 0.6 on QLQ-C30 domain scores. **Table 6.** Regression results (model 6) for EQ-5D-3L based utility values ≥ 0.6 and < 1 on QLQ-C30 domain scores.

**Additional file 3.** Patient characteristics for concurently collected EQ-5D and partially incomplete QLQ-C30 questionnaires for which functional scale scores could still be calcuated.

**Additional file 4: Figure 2.** Predicted EQ-5D-3L utility versus the observed utility for a) the RE model with QLQ-C30 domain scores (preferred model 1); b) the RE model with continuous QLQ-C30 questions (model 2); c) the RE model with QLQ-C30 dummy questions (model 3); d) the ordered logit model on the EQ-5D-3L domains (model 4); e) beta regerssion (model 5) and; f) the separate equations subgroup approach (model 6). **Figure 3.** Prediction error (observed – predicted EQ-5D-3L uility) for a) the RE model with QLQ-C30 domain scores (preferred model 1); b) the RE model with continuous QLQ-C30 questions (model 2); c) the RE model with QLQ-C30 dummy questions (model 3); d) the ordered logit model on the EQ-5D-3L domains (model 4); e) beta regerssion(model 5) and; f) the separate equations subgroup approach (model 6).

## Data Availability

The datasets used and/or analysed during the current study are available from the corresponding author on reasonable request.

## Abbreviations

CAPOX-B: Capecitabine oxaliplatin bevacizumab
CB: Capecitabine bevacizumab
DCCG: Dutch Colorectal Cancer Group
EORTC: European Organisation for Research and Treatmen of Cancer
HRQoL: Health-related quality of life
ICER: Incremental cost-effectiveness ratio
QALY: Quality adjusted life year
mCRC: Metastatic colorectal cancer
OLS: Ordinary least squares
RE: Random effects
RMSE: Root mean square error
MAE: Mean absolute error
U.K.: United Kingdom
