# Supplementary figures and images for: Evaluation of the performance of algorithms mapping EORTC QLQ-C30 onto the EQ-5D index in a metastatic colorectal cancer cost-effectiveness model
Source: Health Qual Life Outcomes. 2020 Jul 20;18:240. doi: 10.1186/s12955-020-01481-2 (PMC7370458; doi:10.1186/s12955-020-01481-2)

**Additional file 1.** Histogram of EQ-5D-3L based utilities of 1905 observations


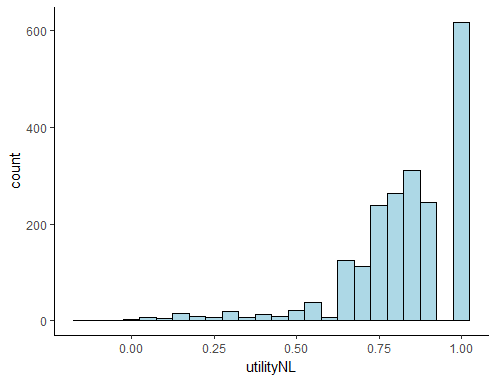

Supplement: Supplementary file 1 — Additional file 1. Histogram of EQ-5D-3L based utilities of 1905 observations. [file 12955_2020_1481_MOESM1_ESM.docx]
