# Supplementary material for: Evaluation of the performance of algorithms mapping EORTC QLQ-C30 onto the EQ-5D index in a metastatic colorectal cancer cost-effectiveness model
Source: Health Qual Life Outcomes. 2020 Jul 20;18:240. doi: 10.1186/s12955-020-01481-2 (PMC7370458; doi:10.1186/s12955-020-01481-2)
Supplement: Supplementary file 2 — Additional file 2: Table 1. Ordered logit regression (model 4) results for QLQ-C30 domain scores on EQ-5D-3L domain. Table 2. Ordered logit regression (model 4) results for QLQ-C30 domain scores on EQ-5D-3L domain. Table 3. Ordered logit regression (model 4) results for QLQ-C30 domain scores on EQ-5D-3L domain. Table 4. Separate equations subgroup approach (model 6) results for QLQ-C30 domain scores on EQ-5D-3L utility of i) < 0.6, ii) ≥ 0.6 and < 1 and iii) 1. Table 5. Regression results (model 6) for EQ-5D-3L based utility values < 0.6 on QLQ-C30 domain scores. Table 6. Regression results (model 6) for EQ-5D-3L based utility values ≥ 0.6 and < 1 on QLQ-C30 domain scores. [file 12955_2020_1481_MOESM2_ESM.docx]

**Additional file 2.**

| Table 1. Ordered logit regression (model 4) results for QLQ-C30 domain scores on EQ-5D-3L domain. | | | | | | |
| --- | --- | --- | --- | --- | --- | --- |
| **Mobility (MO)** | | Coefficient | SD | t-value | p-value* | 95% CI |
|  | Kappa | 8.568 | 0.628 | 13.647 | <0.001 | [7.338,9.799] |
|  | Constant | 9.741 | 0.953 | 10.221 | <0.001 | [7.873,11.609] |
|  | Global health | -0.007 | 0.005 | -1.23 | 0.219 | [-0.017,0.004] |
|  | Physical functioning | -0.093 | 0.007 | -12.5 | <0.001 | [-0.108,-0.078] |
|  | Role functioning | -0.012 | 0.005 | -2.734 | 0.006 | [-0.021,-0.004] |
|  | Emotional functioning | 0.011 | 0.005 | 1.983 | 0.047 | [0,0.021] |
|  | Cognitive functioning | -0.011 | 0.005 | -2.213 | 0.027 | [-0.022,-0.001] |
|  | Social functioning | -0.005 | 0.005 | -1.083 | 0.279 | [-0.015,0.004] |
|  | Symptom scale: fatigue | -0.023 | 0.006 | -4.174 | <0.001 | [-0.034,-0.012] |
|  | Symptom scale: nausea | -0.020 | 0.007 | -2.963 | 0.003 | [-0.033,-0.007] |
|  | Symptom scale: pain | 0.014 | 0.004 | 3.393 | 0.001 | [0.006,0.022] |
|  | Symptom scale: dyspnoea | 0.002 | 0.004 | 0.624 | 0.532 | [-0.005,0.01] |
|  | Symptom scale: insomnia | -0.000 | 0.003 | -0.035 | 0.972 | [-0.007,0.006] |
|  | Symptom scale: appetite loss | -0.001 | 0.004 | -0.204 | 0.838 | [-0.009,0.007] |
|  | Symptom scale: constipation | -0.001 | 0.005 | -0.197 | 0.843 | [-0.01,0.008] |
|  | Symptom scale: diarrhoea | 0.005 | 0.003 | 1.553 | 0.120 | [-0.001,0.012] |
|  | Symptom scale: financial diff. | 0.017 | 0.004 | 3.996 | <0.001 | [0.009,0.025] |
| **Self-care (SC)** | |  | |  |  |  |
|  | Kappa | 3.582 | 0.466 | 7.687 | <0.001 | [2.669,4.495] |
|  | Constant | 2.426 | 1.196 | 2.029 | 0.042 | [0.082,4.77] |
|  | Global health | 0.004 | 0.008 | 0.437 | 0.662 | [-0.013,0.02] |
|  | Physical functioning | -0.056 | 0.010 | -5.887 | <0.001 | [-0.075,-0.037] |
|  | Role functioning | -0.014 | 0.007 | -1.995 | 0.046 | [-0.028,0] |
|  | Emotional functioning | 0.000 | 0.008 | 0.023 | 0.982 | [-0.015,0.015] |
|  | Cognitive functioning | -0.010 | 0.007 | -1.304 | 0.192 | [-0.024,0.005] |
|  | Social functioning | 0.004 | 0.007 | 0.604 | 0.546 | [-0.01,0.019] |
|  | Symptom scale: fatigue | -0.008 | 0.008 | -0.935 | 0.350 | [-0.024,0.009] |
|  | Symptom scale: nausea | -0.002 | 0.009 | -0.196 | 0.845 | [-0.02,0.016] |
|  | Symptom scale: pain | 0.011 | 0.005 | 2.046 | 0.041 | [0,0.022] |
|  | Symptom scale: dyspnoea | -0.006 | 0.006 | -1.005 | 0.315 | [-0.017,0.005] |
|  | Symptom scale: insomnia | 0.008 | 0.005 | 1.641 | 0.101 | [-0.002,0.017] |
|  | Symptom scale: appetite loss | -0.007 | 0.006 | -1.114 | 0.265 | [-0.018,0.005] |
|  | Symptom scale: constipation | -0.010 | 0.006 | -1.575 | 0.115 | [-0.023,0.002] |
|  | Symptom scale: diarrhoea | 0.010 | 0.005 | 2.020 | 0.043 | [0,0.021] |
|  | Symptom scale: financial diff. | 0.015 | 0.005 | 2.761 | 0.006 | [0.004,0.026] |

p-values result from a t-test.

| Table 2. Ordered logit regression (model 4) results for QLQ-C30 domain scores on EQ-5D-3L domain. | | | | | | |
| --- | --- | --- | --- | --- | --- | --- |
| **Daily activities (DA)** | | Coefficient | SD | t-value | p-value* | 95% CI |
|  | Kappa | 6.652 | 0.333 | 19.977 | <0.001 | [6,7.305] |
|  | Constant | 9.823 | 0.957 | 10.267 | <0.001 | [7.948,11.698] |
|  | Global health | -0.014 | 0.006 | -2.427 | 0.015 | [-0.025,-0.003] |
|  | Physical functioning | -0.032 | 0.007 | -4.613 | <0.001 | [-0.045,-0.018] |
|  | Role functioning | -0.062 | 0.005 | -12.095 | <0.001 | [-0.072,-0.052] |
|  | Emotional functioning | 0.006 | 0.006 | 0.999 | 0.318 | [-0.005,0.017] |
|  | Cognitive functioning | -0.018 | 0.005 | -3.361 | 0.001 | [-0.029,-0.008] |
|  | Social functioning | -0.015 | 0.005 | -3.039 | 0.002 | [-0.025,-0.005] |
|  | Symptom scale: fatigue | 0.021 | 0.006 | 3.654 | <0.001 | [0.01,0.033] |
|  | Symptom scale: nausea | -0.006 | 0.007 | -0.918 | 0.359 | [-0.019,0.007] |
|  | Symptom scale: pain | 0.008 | 0.004 | 2.007 | 0.045 | [0,0.017] |
|  | Symptom scale: dyspnoea | 0.001 | 0.004 | 0.186 | 0.852 | [-0.007,0.008] |
|  | Symptom scale: insomnia | 0.003 | 0.003 | 0.972 | 0.331 | [-0.003,0.01] |
|  | Symptom scale: appetite loss | 0.003 | 0.004 | 0.752 | 0.452 | [-0.005,0.011] |
|  | Symptom scale: constipation | 0.002 | 0.005 | 0.459 | 0.646 | [-0.007,0.011] |
|  | Symptom scale: diarrhoea | 0.003 | 0.004 | 0.838 | 0.402 | [-0.004,0.011] |
|  | Symptom scale: financial diff. | 0.009 | 0.004 | 2.181 | 0.030 | [0.001,0.018] |
| **Pain and discomfort (PA)** | |  | |  |  |  |
|  | Kappa | 8.559 | 0.506 | 16.929 | <0.001 | [7.568,9.55] |
|  | Constant | 0.262 | 0.869 | 0.302 | 0.763 | [-1.441,1.964] |
|  | Global health | -0.009 | 0.006 | -1.5 | 0.134 | [-0.02,0.003] |
|  | Physical functioning | -0.009 | 0.007 | -1.346 | 0.178 | [-0.023,0.004] |
|  | Role functioning | 0.005 | 0.005 | 1.102 | 0.270 | [-0.004,0.015] |
|  | Emotional functioning | -0.004 | 0.006 | -0.682 | 0.495 | [-0.015,0.007] |
|  | Cognitive functioning | -0.007 | 0.005 | -1.287 | 0.198 | [-0.018,0.004] |
|  | Social functioning | -0.002 | 0.005 | -0.354 | 0.723 | [-0.012,0.009] |
|  | Symptom scale: fatigue | 0.003 | 0.006 | 0.567 | 0.571 | [-0.008,0.015] |
|  | Symptom scale: nausea | -0.023 | 0.007 | -3.182 | 0.001 | [-0.037,-0.009] |
|  | Symptom scale: pain | 0.109 | 0.006 | 17.809 | <0.001 | [0.097,0.121] |
|  | Symptom scale: dyspnoea | 0.007 | 0.004 | 1.657 | 0.098 | [-0.001,0.015] |
|  | Symptom scale: insomnia | 0.005 | 0.003 | 1.509 | 0.131 | [-0.002,0.012] |
|  | Symptom scale: appetite loss | -0.009 | 0.004 | -2.114 | 0.034 | [-0.018,-0.001] |
|  | Symptom scale: constipation | 0.014 | 0.005 | 3.045 | 0.002 | [0.005,0.024] |
|  | Symptom scale: diarrhoea | 0.005 | 0.004 | 1.306 | 0.191 | [-0.003,0.013] |
|  | Symptom scale: financial diff. | -0.000 | 0.005 | -0.008 | 0.994 | [-0.009,0.009] |

p-values result from a t-test.

| Table 3. Ordered logit regression (model 4) results for QLQ-C30 domain scores on EQ-5D-3L domain. | | | | | | |
| --- | --- | --- | --- | --- | --- | --- |
| **Anxiety and depression (AD)** | | Coefficient | SD | t-value | p-value* | 95% CI |
|  | Kappa | 5.685 | 0.390 | 14.568 | <0.001 | [4.92,6.45] |
|  | Constant | 11.065 | 1.033 | 10.715 | <0.001 | [9.041,13.089] |
|  | Global health | -0.007 | 0.006 | -1.057 | 0.291 | [-0.019,0.006] |
|  | Physical functioning | -0.005 | 0.007 | -0.742 | 0.458 | [-0.02,0.009] |
|  | Role functioning | -0.009 | 0.005 | -1.79 | 0.073 | [-0.019,0.001] |
|  | Emotional functioning | -0.123 | 0.007 | -16.542 | <0.001 | [-0.138,-0.109] |
|  | Cognitive functioning | -0.002 | 0.005 | -0.355 | 0.722 | [-0.013,0.009] |
|  | Social functioning | -0.003 | 0.005 | -0.556 | 0.578 | [-0.013,0.007] |
|  | Symptom scale: fatigue | -0.005 | 0.006 | -0.813 | 0.416 | [-0.018,0.007] |
|  | Symptom scale: nausea | 0.005 | 0.007 | 0.745 | 0.457 | [-0.008,0.019] |
|  | Symptom scale: pain | -0.011 | 0.004 | -2.437 | 0.015 | [-0.02,-0.002] |
|  | Symptom scale: dyspnoea | -0.009 | 0.004 | -2.074 | 0.038 | [-0.018,0] |
|  | Symptom scale: insomnia | 0.011 | 0.004 | 3.179 | 0.001 | [0.004,0.018] |
|  | Symptom scale: appetite loss | -0.000 | 0.004 | -0.024 | 0.981 | [-0.009,0.008] |
|  | Symptom scale: constipation | -0.008 | 0.005 | -1.714 | 0.087 | [-0.018,0.001] |
|  | Symptom scale: diarrhoea | -0.005 | 0.004 | -1.095 | 0.274 | [-0.013,0.004] |
|  | Symptom scale: financial diff. | 0.007 | 0.004 | 1.656 | 0.098 | [-0.001,0.016 |

p-values result from a t-test.

Utilities can be calculated by applying the composing equations for each EQ-5D domain :

In example for EQ-5D domain mobility:

MO = 9.740831 -0.00654**global health score* -0.09308* *physical functioning score* -0.01237* *role functioning score* +0.010595* *emotional functioning score* -0.01149* *cognitive functioning score* -0.00532* *social functioning score* -0.02323**fatigue score* -0.01976**nausea score* +0.014166**pain score* +0.002403**dyspnoea score* -0.00011**insomnia score* -0.00083**appetite score* -0.00088**constipation score* +0.005426**diarrhoea score* +0.016817**financial difficulties score*

probmo1 = 1/(1 + exp(MO))

probmo2 = 1/(1 + exp(MO - kappa)) – 1/(1+exp(MO))

probmo3= 1-Probmo1-Promo2

Where prob stands for the predicted probability of the EQ-5D-3L response level (1, 2 or 3). Thus probmo1 stands for the probability of a level 1 response for the EQ-5D mobility domain. Probmo2 and probmo3 for level 2 and level 3 responses on the EQ-5D mobility domain respectively . Each probability for each EQ-5D domain can be used for EQ-5D tariff calculations, in example for the Dutch tariff:

Estimated EQ-5D=1-(probmo2*0.036)-(probmo3*0.161)-(probsc2*0.082)-(probsc3*0.152)-(probda2*0.032)-(probda3*0.057)-(probpa2*0.086)-(probpa3*0.329)-(probad2*0.124)-(probad3*0.325)-(1-ProbPerfect)*0.071-ProbN3*0.234

ProbPerfect= probmo1*probsc1*probda1*probpa1*probad1

ProbN3= 1-(1-probmo3)*(1-probsc3)*(1-probda3)*(1-probpa3)*(1-probad3)

| Table 4. Separate equations subgroup approach (model 6) results for QLQ-C30 domain scores on EQ-5D-3L utility of i) < 0.6, ii) $\geq$ 0.6 and < 1 and iii) 1. | | | | | | |
| --- | --- | --- | --- | --- | --- | --- |
| Variable | | Coefficient | SD | t-value | p-value* | 95% CI |
| **Category 1: utility < 0.6** | | (Base outcome) | | | | |
| **Category 2: utility** $\boldsymbol{\geq}$ **0.6 and < 1** | |  | |  |  |  |
|  | Constant | -4.355 | 1.305 | -3.338 | 0.001 | [-6.913,-1.798] |
|  | Global health | 0.011 | 0.010 | 1.152 | 0.249 | [-0.008,0.03] |
|  | Physical functioning | 0.044 | 0.010 | 4.229 | <0.001 | [0.024,0.064] |
|  | Role functioning | 0.022 | 0.008 | 2.684 | 0.007 | [0.006,0.038] |
|  | Emotional functioning | 0.031 | 0.008 | 3.771 | <0.001 | [0.015,0.048] |
|  | Cognitive functioning | 0.008 | 0.008 | 1.015 | 0.310 | [-0.007,0.023] |
|  | Social functioning | -0.002 | 0.008 | -0.283 | 0.777 | [-0.017,0.013] |
|  | Symptom scale: fatigue | -0.013 | 0.009 | -1.364 | 0.173 | [-0.031,0.005] |
|  | Symptom scale: nausea | -0.006 | 0.009 | -0.656 | 0.512 | [-0.023,0.011] |
|  | Symptom scale: pain | -0.023 | 0.006 | -3.999 | <0.001 | [-0.034,-0.012] |
|  | Symptom scale: dyspnoea | 0.006 | 0.006 | 1.016 | 0.310 | [-0.006,0.018] |
|  | Symptom scale: insomnia | -0.006 | 0.005 | -1.181 | 0.238 | [-0.016,0.004] |
|  | Symptom scale: appetite loss | 0.004 | 0.006 | 0.641 | 0.521 | [-0.008,0.016] |
|  | Symptom scale: constipation | 0.008 | 0.007 | 1.212 | 0.225 | [-0.005,0.021] |
|  | Symptom scale: diarrhoea | 0.000 | 0.006 | -0.054 | 0.957 | [-0.012,0.011] |
|  | Symptom scale: financial diff. | -0.012 | 0.007 | -1.870 | 0.062 | [-0.025,0.001] |
| **Category 3: utility = 1** | |  | |  |  |  |
|  | Constant | -18.864 | 1.994 | -9.461 | <0.001 | [-22.772,-14.956] |
|  | Global health | 0.024 | 0.012 | 2.046 | 0.041 | [0.001,0.048] |
|  | Physical functioning | 0.116 | 0.015 | 7.926 | <0.001 | [0.087,0.145] |
|  | Role functioning | 0.046 | 0.010 | 4.470 | <0.001 | [0.026,0.066] |
|  | Emotional functioning | 0.055 | 0.012 | 4.748 | <0.001 | [0.032,0.077] |
|  | Cognitive functioning | 0.028 | 0.011 | 2.579 | 0.010 | [0.007,0.05] |
|  | Social functioning | 0.009 | 0.011 | 0.890 | 0.373 | [-0.011,0.03] |
|  | Symptom scale: fatigue | -0.021 | 0.011 | -1.866 | 0.062 | [-0.044,0.001] |
|  | Symptom scale: nausea | 0.022 | 0.014 | 1.628 | 0.103 | [-0.005,0.049] |
|  | Symptom scale: pain | -0.107 | 0.012 | -9.208 | <0.001 | [-0.13,-0.085] |
|  | Symptom scale: dyspnoea | 0.006 | 0.008 | 0.744 | 0.457 | [-0.01,0.022] |
|  | Symptom scale: insomnia | -0.024 | 0.007 | -3.459 | 0.001 | [-0.038,-0.011] |
|  | Symptom scale: appetite loss | 0.012 | 0.009 | 1.360 | 0.174 | [-0.005,0.029] |
|  | Symptom scale: constipation | -0.001 | 0.010 | -0.095 | 0.924 | [-0.02,0.018] |
|  | Symptom scale: diarrhoea | -0.001 | 0.008 | -0.149 | 0.882 | [-0.016,0.014] |
|  | Symptom scale: financial diff. | -0.037 | 0.010 | -3.890 | <0.001 | [-0.056,-0.018] |

p-values result from a t-test.

| Table 5. Regression results (model 6) for EQ-5D-3L based utility values < 0.6 on QLQ-C30 domain scores. | | | | | |
| --- | --- | --- | --- | --- | --- |
| Variable | Coefficient | SD | t-value | p-value* | 95% CI |
| Constant | 0.313 | 0.054 | 5.826 | <0.001 | [0.208,0.418] |
| Emotional functioning | 0.002 | 0.001 | 3.158 | 0.002 | [0.001,0.003] |
| Symptom scale: pain | -0.001 | 0.001 | -2.621 | 0.010 | [-0.002,0] |

p-values result from a t-test.

| Table 6. Regression results (model 6) for EQ-5D-3L based utility values $\geq$ 0.6 and < 1 on QLQ-C30 domain scores. | | | | | |
| --- | --- | --- | --- | --- | --- |
| Variable | Coefficient | SD | t-value | p-value* | 95% CI |
| Constant | 0.570 | 0.017 | 33.378 | <0.001 | [0.536,0.603] |
| Physical functioning | 0.001 | 0.000 | 3.983 | <0.001 | [0,0.001] |
| Role functioning | 0.000 | 0.000 | 2.373 | 0.018 | [0,0.001] |
| Emotional functioning | 0.002 | 0.000 | 13.325 | <0.001 | [0.002,0.002] |
| Symptom scale: pain | -0.001 | 0.000 | -8.869 | <0.001 | [-0.001,-0.001] |
| Symptom scale: insomnia | 0.000 | 0.000 | -1.902 | 0.057 | [0,0] |

p-values result from a t-test.
