# Supplementary material for: Evaluation of the performance of algorithms mapping EORTC QLQ-C30 onto the EQ-5D index in a metastatic colorectal cancer cost-effectiveness model
Source: Health Qual Life Outcomes. 2020 Jul 20;18:240. doi: 10.1186/s12955-020-01481-2 (PMC7370458; doi:10.1186/s12955-020-01481-2)
Supplement: Supplementary file 3 — Additional file 3. Patient characteristics for concurently collected EQ-5D and partially incomplete QLQ-C30 questionnaires for which functional scale scores could still be calcuated. [file 12955_2020_1481_MOESM3_ESM.docx]

**Additional file 3.** Patient characteristics for concurently collected EQ-5D and partially incomplete QLQ-C30 questionnaires for which functional scale scores could still be calcuated.

|  |  | N = 120 |
| --- | --- | --- |
| Age (years) |  | 66 (8.3) |
| Male gender (%) |  | 60 |
| EQ-5D-3L* | N | 120 |
|  | Mobility 1/2/3 (%) | 40.8/55/4.2 |
|  | Self-cae 1/2/3 (%) | 83.3/13.3/3.3 |
|  | Usual activities 1/2/3 (%) | 45.8/47.5/6.7 |
|  | Pain/discomfort 1/2/3 (%) | 46.7/48.3/5 |
|  | Depression/anxiety 1/2/3 (%) | 72.5/26.7/0.8 |
|  | EQ-5D utility, mean (SD) | 0.76 (0.232) |
|  | EQ-5D range | -0.086 to 1 |
| QLQ-C30 v.3.0 | Questionnaires, N | 120 |
|  | Physical functioning, mean (SD) | 74.801 (22.661) |
|  | Role functioning, mean (SD) | 69.861 (28.113) |
|  | Emotional functioning, mean (SD) | 80.949 (18.591) |
|  | Cognitive functioning, mean (SD) | 86.111 (18.629) |
|  | Social functioning, mean (SD) | 85.139 (20.245) |
|  | Global health, mean (SD) | 68.75 (20.247) |
|  | Fatigue, mean (SD) | 30.972 (22.942) |
|  | Nausea/vomiting, mean (SD) | 6.389 (13.351) |
|  | Pain, mean (SD) | 25.556 (28.903) |
|  | Dyspnea, mean (SD) | 16.111 (25.559) |
|  | Insomnia, mean (SD) | 19.444 (26.137) |
|  | Appetite, mean (SD) | 14.167 (22.314) |
|  | Constipation, mean (SD) | 5.278 (12.961) |
|  | Diarrhea, mean (SD) | 11.667 (22.723) |
|  | Financial difficulties, mean (SD) | 4.167 (13.363) |

* Percentages at level 1, 2 and 3 represent no problems at all, some problems and extreme problems, respectively.
