# Supplementary material for: Evaluation of the performance of algorithms mapping EORTC QLQ-C30 onto the EQ-5D index in a metastatic colorectal cancer cost-effectiveness model
Source: Health Qual Life Outcomes. 2020 Jul 20;18:240. doi: 10.1186/s12955-020-01481-2 (PMC7370458; doi:10.1186/s12955-020-01481-2)
Supplement: Supplementary file 4 — Additional file 4: Figure 2. Predicted EQ-5D-3L utility versus the observed utility for a) the RE model with QLQ-C30 domain scores (preferred model 1); b) the RE model with continuous QLQ-C30 questions (model 2); c) the RE model with QLQ-C30 dummy questions (model 3); d) the ordered logit model on the EQ-5D-3L domains (model 4); e) beta regerssion (model 5) and; f) the separate equations subgroup approach (model 6). Figure 3. Prediction error (observed – predicted EQ-5D-3L uility) for a) the RE model with QLQ-C30 domain scores (preferred model 1); b) the RE model with continuous QLQ-C30 questions (model 2); c) the RE model with QLQ-C30 dummy questions (model 3); d) the ordered logit model on the EQ-5D-3L domains (model 4); e) beta regerssion(model 5) and; f) the separate equations subgroup approach (model 6). [file 12955_2020_1481_MOESM4_ESM.docx]

**Additional file 4.**

| 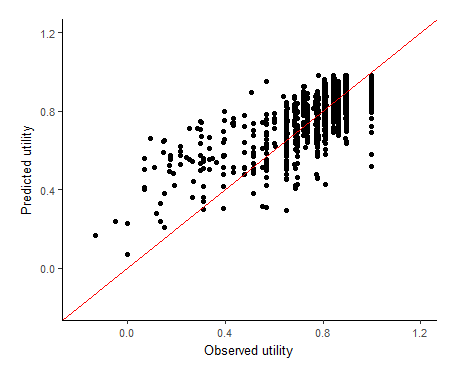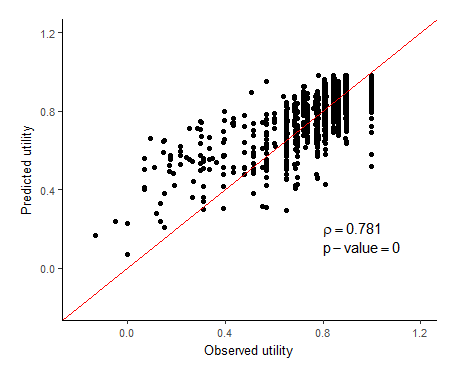a) | 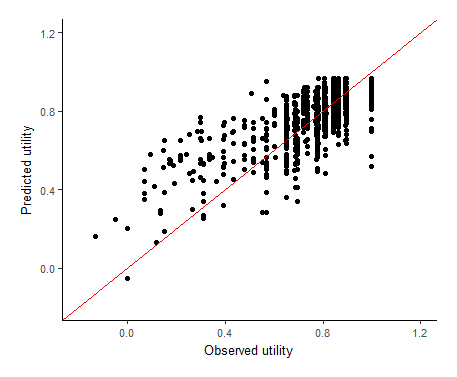b) |
| --- | --- |
| 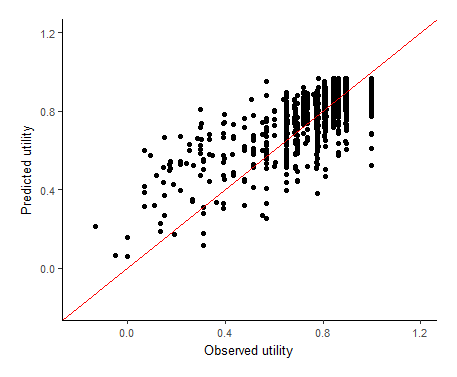c) | 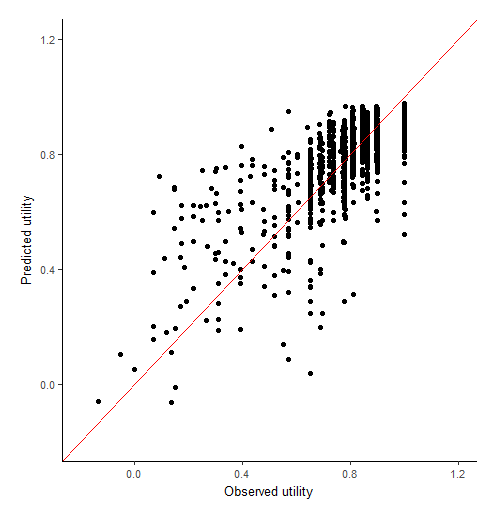d) |
| 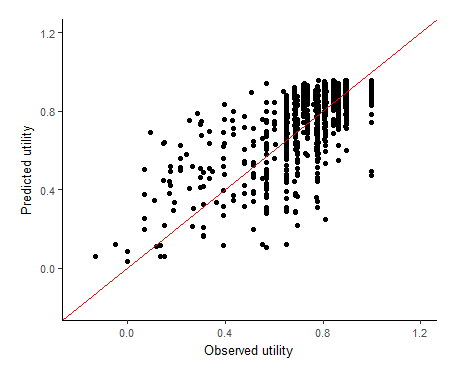e) | 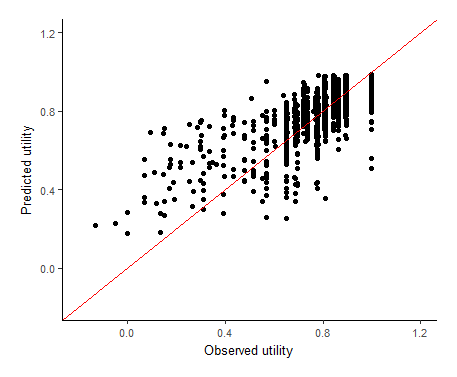f) |
| Figure 2. Predicted EQ-5D-3L utility versus the observed utility for a) the RE model with QLQ-C30 domain scores (preferred model 1); b) the RE model with continuous QLQ-C30 questions (model 2); c) the RE model with QLQ-C30 dummy questions (model 3); d) the ordered logit model on the EQ-5D-3L domains (model 4); e) beta regerssion (model 5) and; f) the separate equations subgroup approach (model 6). | |
| 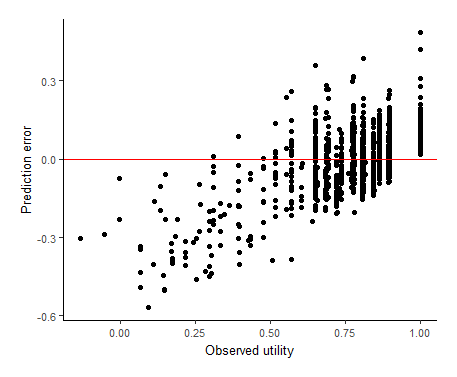a) | 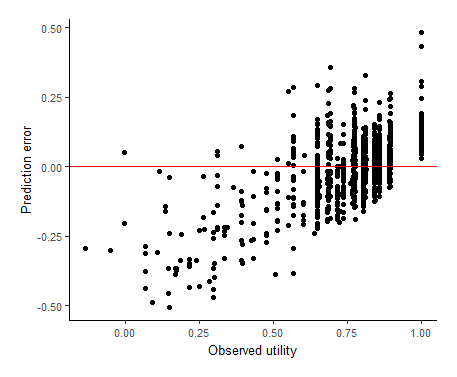b) |
| 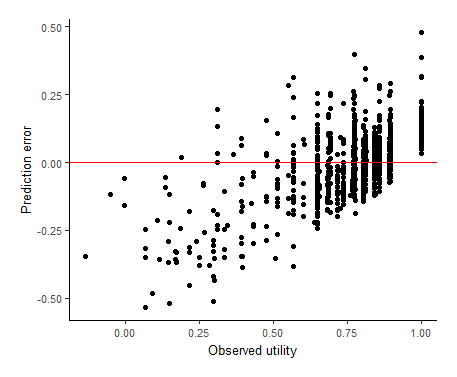c) | 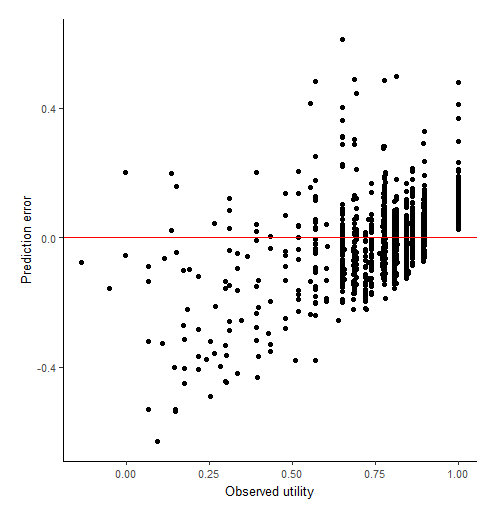d) |
| 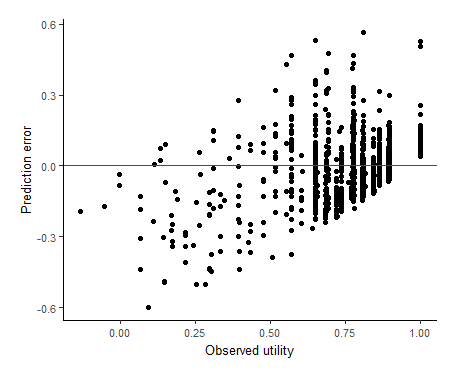e) | 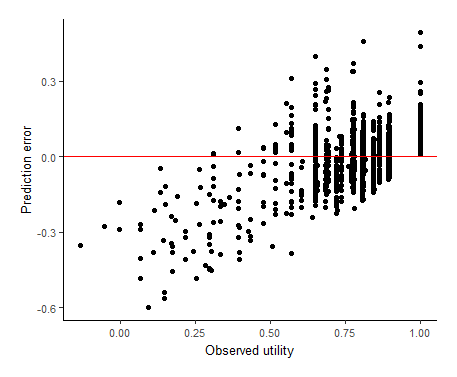f) |
| Figure 3. Prediction error (observed – predicted EQ-5D-3L uility) for a) the RE model with QLQ-C30 domain scores (preferred model 1); b) the RE model with continuous QLQ-C30 questions (model 2); c) the RE model with QLQ-C30 dummy questions (model 3); d) the ordered logit model on the EQ-5D-3L domains (model 4); e) beta regerssion(model 5) and; f) the separate equations subgroup approach (model 6). | |
